# An arrowhead found incidentally in the chest during workup for unrelated disease after 22-years of initial injury

**DOI:** 10.4103/0974-2700.76819

**Published:** 2011

**Authors:** Shilpi Singh Gupta, Onkar Singh, Sumit Shukla, Raj Kumar Mathur

**Affiliations:** Department of Surgery, MGM Medical College and MY Hospital, Indore - 452 001, India

Sir,

Recently, we cared for an interesting case that we would like to share with the readers of this esteemed journal. A 68-year-old male who belonged to a tribal area in the central India, presented in the emergency room with features of intestinal perforation. He was immediately admitted and preparation started for emergency laparotomy. During workup, chest radiograph was ordered, which showed surprising findings. An arrowhead directed upside down was lying in the left chest cavity. There was no evidence of pneumothorax or hydrothorax. On careful history taking, the patient admitted that he was struck with an arrow, 22-years back when the neighboring tribe attacked his tribe. He remembered that the arrow penetrated into his chest in a direction parallel to the neck on left side when he was lying on the ground for hiding, but told that he had pulled it out and thrown it away. He had never been aware of the fact that the arrowhead had been retained inside. It had never caused him any problem since then.

**Figure 1:**
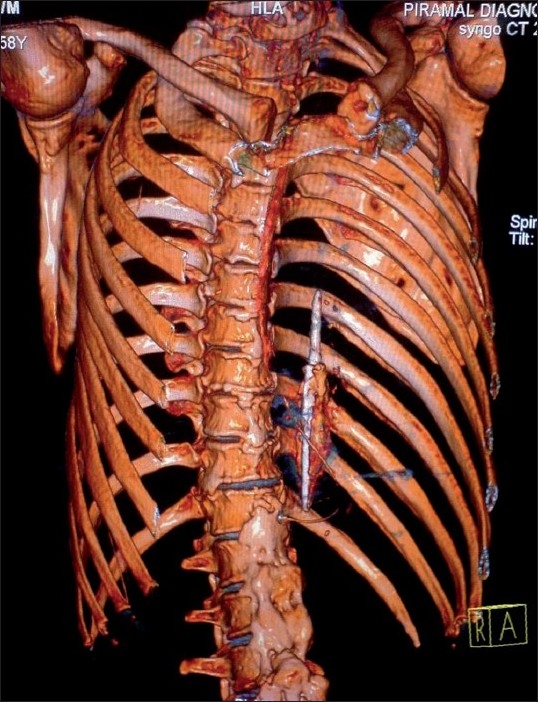
Three-dimensional virtual CT scan image showing arrowhead in the left chest cavity

**Figure 2:**
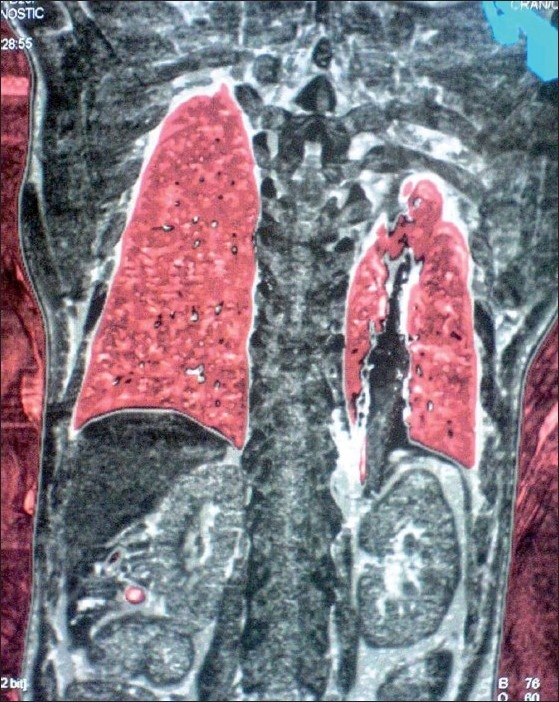
Three-dimensional virtual CT scan image showing the track followed by the arrowhead that now lies with its tip just in touch with upper and middle pole of left kidney. It has not caused any pneumo/hemothorax

Computerized Tomography (CT) scan was ordered immediately to assess the exact situation. Three-dimensional virtual images were constructed using the raw data from the CT scan. These images revealed that the arrowhead [Figure 1] had traveled from its entry point at the left supraclavicular fossa (as told by the patient) to a point with its tip just touching the upper and medial pole of the left kidney [Figure 2]. The movement of the arrowhead must have been extremely slow, and it had left behind a track that was visible on CT scan [Figure 2]. As the arrowhead was lying inside for 22 years without causing any problem, and the patient was already in seventh decade of life, we planned to leave the arrowhead as it was. He was operated for perforation peritonitis and discharged subsequently. The arrowhead has been lying inside his body for 1 year without causing any problem.

Arrow injuries are infrequent now days, and in developed countries, these are considered an extinct form of injury.[1,2] However, such cases are seen by surgeons practicing in parts of India that cover the areas which home many tribes. Therefore, the arrow injuries are still relevant in our country.[2] Such injuries usually result from communal conflicts. Presentation is acute in almost 100% cases.[3] Arrow actually penetrates the tissues and bones to enter the body cavities and can injure the heart or vessels. Depending on the arrowhead and the tissue elasticity, variable degrees of tamponade occurs due to the arrow present within the wound. Thus, an arrow should not be extracted before any injury to the hilum or the heart is ruled out.[4] Arrows have lesser velocity, and thus cause less destructive injuries (barbed arrows cause more damage when removed) than those caused by bullets.[5]

Although, vital stability favors no major vital injury, but if doubt about any such injury exists, patient needs to be investigated. In such situations, spiral CT with contrast and two-dimensional image reconstruction has been recommended.[4] CT can also pick subtle hematomas that are usually missed on angiogram. Even if no major injuries are present, a chest tube should be inserted before removing the arrow (if not has already been inserted upon arrival).[4] In the case of vascular/heart injury or hemodynamic instability, successful treatment is guided by optimal exposure, adequate mobilization, minimizing bleeding, and good repair.[2,4]

We searched the literature using PubMed and other search engines but could not find any similar case. This case emphasizes that sometimes arrow injury to the chest may remain asymptomatic and go unnoticed even for years. The arrowhead if not removed and retained inside may travel away from the site of entry. We might have considered removal of the arrowhead had the patient been younger or came with some complication because of it. Recently, a case of a patient has been reported who survived 3 days after such an injury and traveled 1000 km for treatment with an arrow pulsating with the heart inside the chest.[6]
